# Microbial Diversity and Pathogenic Properties of Microbiota Associated with Aerobic Vaginitis in Women with Recurrent Pregnancy Loss

**DOI:** 10.3390/diagnostics12102444

**Published:** 2022-10-09

**Authors:** Karima Ncib, Wael Bahia, Nadia Leban, Abdelkarim Mahdhi, Fatma Trifa, Ridha Mzoughi, Anis Haddad, Chédia Jabeur, Gilbert Donders

**Affiliations:** 1 Laboratory of Analysis, Treatment and Valorization of Pollutants of the Environment and Products (LATVPEP), Faculty of Pharmacy, University of Monastir, Monastir 5000, Tunisia; 2 Research Unit of Clinical and Molecular Biology, Department of Biochemistry, Faculty of Pharmacy, University of Monastir, Monastir 5000, Tunisia; 3 Laboratory of Human Genome and Multifactorial Diseases, Faculty of Pharmacy of Monastir, University of Monastir, Monastir 5000, Tunisia; 4 Higher Institute of Biotechnology of Monastir, Monastir 5000, Tunisia; 5 Department of Obstetrics and Gynecology, Fattouma Bourguiba University Hospital, Monastir 5000, Tunisia; 6 Femicare VZW Clinical Research for Women, 3300 Tienen, Belgium; 7 Department Obstetrics and Gynecology, University Hospital Antwerp, 2650 Edegem, Belgium; 8 President of the International Society for Infectious Diseases (ISIDOG), 3300 Tienen, Belgium

**Keywords:** aerobic vaginitis, recurrent pregnancy loss, antibiotic resistance, biofilm formation, dysbiosis, *lactobacillus*, enteric bacteria, preterm birth, miscarriage, chorioamnionitis

## Abstract

Recurrent pregnancy loss (RPL) is a major reproductive problem that affects approximately 5% of couples. The objective of this study was to assess vaginal flora dysbiosis in women suffering from unexplained RPL and to investigate the pathogenic properties of the microbiota associated with aerobic vaginitis (AV). The study included one hundred fifteen women, 65 with RPL and 50 controls. The diversity of vaginal microbiota isolated was evaluated by molecular sequencing. Then, pathogenic factors, such as acid-resistance, antibiotics susceptibility, and biofilm formation were evaluated. The prevalence of AV was five-fold higher in the RPL group than in the controls (64.6% vs. 12.0%). The most prevalent isolates in the case group were *Enterococcus* spp. (52%) and *Staphylococcus* spp. (26%). All bacterial strains tolerate low pH. The prevalence of multidrug resistance (MDR) among all bacteria was 47.7%. Of all strains, 91.0% were biofilm producers. The presence of MDR was found to be related to biofilm formation. The results provide evidence supporting an increased presence of dysbiosis of the vaginal flora, especially AV, in women with RPL in Tunisia. The viability of the AV-associated bacteria and their persistence in the genitals may be due to their ability to resist low pH and to produce a biofilm.

## 1. Introduction

Recurrent pregnancy loss (RPL) is defined as at least two failed clinical pregnancies before 20 weeks post-menstruation [1,2]. It is considered a major reproductive problem, affecting approximately five-percent of couples trying to conceive [3]. In fact, even while the conditions for conception are ideal, only about one-third of all pregnancies evolve into successful pregnancies [4]. RPL has a diverse etiology. However, in the vast majority of cases (50–70%) the pathophysiology remains unexplained [5]. One underexplored and underestimated risk factor is the presence of vaginal dysbiosis during pregnancy. Early pregnancy loss can be caused by ascending infection from the lower genital tract through the cervical canal resulting embryo–fetal infection [6,7], but also other mechanisms, such as the production of pro-inflammatory and cervix-weakening substances in the vagina can play a role [6]. To understand the pathological events related to ascending infection from the vagina, it is helpful to understand the normal vaginal microbiome. In normal vaginal microflora, the ecosystem is made up of diverse microorganisms coexisting in a dynamic balance and establishing complex connections with each other and with the host. Generally, the vaginal microbiome shows a dominance of lactobacillus [8]. lactobacillus species protect the vaginal flora from genital pathogens by producing lactic acid, H_2_O_2_, and antimicrobial proteins. In case of a decrease in the number of lactobacillus species, these can be replaced by anaerobic and/or facultative aerobic microorganisms [9]. The most common cases of dysbiosis are caused by bacterial vaginosis (BV). BV has a high concentration of mixed flora of aerobic, anaerobic, and microaerophilic species [10], typically without causing an inflammatory response. While BV is a well-known type of vaginal dysbiosis, aerobic vaginitis (AV) is an more inflammatory dysbiotic condition that remains largely understudied and often misdiagnosed [11]. AV is also frequently found in reproductive-age women [12] and harbors facultative anaerobic, enteric, or aerobic bacteria, such as *Staphylococcus aureus*, *group B streptococcus*, *E.coli*, *Enterococcus faecalis*, and *Klebsiella* spp. [13,14], but *Prevotella* spp. is also commonly encountered [15]. As these are pro-inflammatory micro-organisms, AV should also be considered as a potential contributing factor of maternofetal infection in addition to BV [16,17,18]. There is increasing evidence that in maternal–fetal health, a crucial role is played by the vaginal bacterial composition. [19,20]. Recently, embryo–fetal bacterial infections have been reported to cause recurrent spontaneous miscarriage [21,22].

Especially early on in pregnancy, both BV and AV seem to be associated with spontaneous miscarriage [14,23,24]. Despite this firm association of vaginal dysbiosis with recurrent miscarriage (RM), the pathophysiologic mechanisms of this relationship are only poorly understood [25]. Different studies have been conducted to assess the relationship between BV and a history of spontaneous abortions [10,26,27]; however, very few studies have focused on the role of AV, bacterial virulence, resistance to antibiotics, pH, and bacterial biofilm formation in the pathogenesis of RPL. In the current study, we focused the research on the implication of AV on RPL. To our knowledge, this is the first report addressing the relationship between aerobic vaginitis disorders and the history of recurrent pregnancy loss. To better understand the relationship between vaginal microbiome alterations and the history of recurrent pregnancy loss, we used Gram staining, culture, and molecular identification of vaginal fluid. To gain new insight into the persistence of the AV-associated bacteria in the genital tract, we evaluated the growth potential of the isolates in a varying pH environment, their level of resistance to different antibiotics, and the potential of biofilm formation.

## 2. Materials and Methods

### 2.1. Study Area and Population

From June 2018 to May 2019, all women with a history of RPL presenting at the Neonatal and Maternity Center in Monastir (Tunisia) were enrolled. The study included 115 women. Sixty-five had suffered from unexplained RPL (cases), and 50 non-pregnant women presenting for a routine checkup visit were randomly selected. Initially, we planned a 1:1 ratio, with 50 cases and an equal number of controls. We aimed to match cases with an equal number of women with the same age and parity as controls. With this intention, during the study period, we came across 65 cases with RPL in their history, but only came across 50 matching controls who wanted to consent. Control-group women had to be non-pregnant, presenting for a routine checkup visit following a case, with a history of at least two successful pregnancies without miscarriages, and were to fall within the age and parity range of the preceding cases.

### 2.2. Inclusion and Exclusion Criteria

Cases were asked for their participation if they had experienced two or more pregnancy losses before 20 weeks of pregnancy. Women who were currently pregnant or menopausal were excluded. If a known cause of RPL (see exclusion criteria listed below) was present, or if it had occurred in a pregnancy obtained by medically assisted fertilization techniques, patients were not included. The presence of symptoms of vulvovaginitis or other vaginal disorder symptoms was recorded, but was not an exclusion criterion. Further exclusion criteria were: use of any antibiotics in the past month, use of vaginal douches or topical agents in the last two weeks, the presence of genital pathology such as fibroids, endometrial polyps, (history of) uterine abnormalities, uncontrolled chronic diseases (e.g., diabetes, hormonal or metabolic disorders, autoimmune disorders, and malignancies), and pregnancies despite severe male-factor infertility.

### 2.3. Ethical Approval and Sample Collection

The protocol of this retrospective study was approved by the ethics committee of the Faculty of Medicine, Monastir, Tunisia (1807–2018, July 2018). After insertion of a sterilized unlubricated speculum, excessive vaginal secretions were cleansed by cotton buds slightly moistened in an appropriate but small amount of saline solution. From the high lateral vaginal border, two vaginal swabs were collected. The first one was immediately placed in Amies’ modified Stuart medium (Oxoid, Hampshire, UK) for microbiological culture in laboratory. The second one served for wet mount and Gram-stained smears.

### 2.4. Diagnosis of Bacterial Disorder Using Wet Mount and Gram Stain of Vaginal Smears

**Figure 1:**
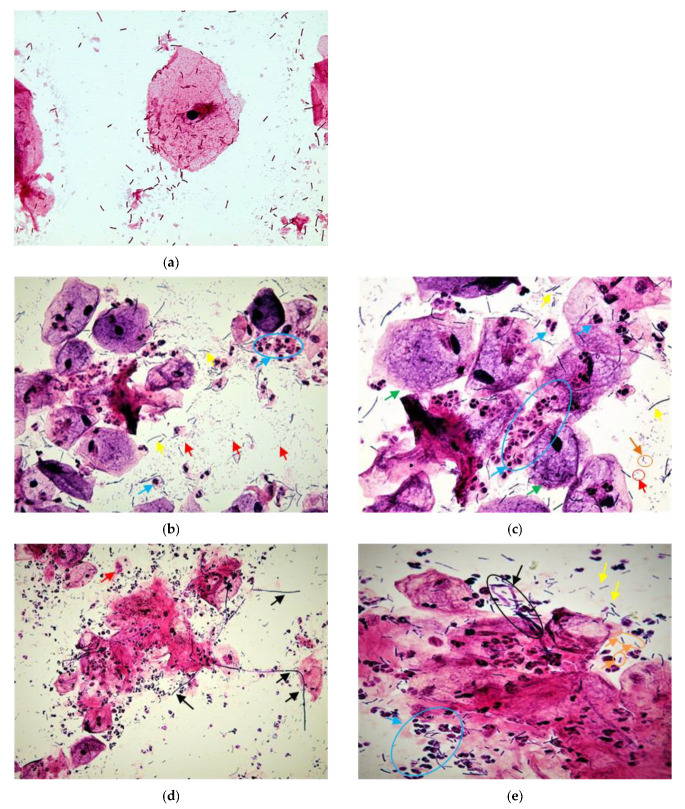
Microscopic images of vaginal smears after Gram staining. (**a**) Normal vaginal flora, a typical superficial cell, and healthy-appearing lactobacillus flora without leukocyte response. (**b**) Typical aerobic vaginitis flora (AV). Phase-contrast image in 400× magnification. (**c**) AV image in 1000× magnification. The yellow arrows indicate *Lactobacilli* spp. bacteria grade II_b_. The red arrows indicate the presence of small bacilli (Enterobacteria-like bacteria) or orange circle shows cocci in pairs. The blue arrows indicate leukocytes in toxic form (+10/cell). The green arrows indicated clue cells. Bacteria cover superficial epithelial cells and give them a specific blurry appearance. (**d**) AV mixed with candidosis; phase-contrast image in 400× magnification. (**e**) Image in 1000× magnification. The yellow arrows indicate *Lactobacilli* spp. bacteria; orange arrows and circle indicate cocci in pairs or in chain. The blue arrows indicate leukocytes in toxic form (+10/cell). The red arrows indicate cytolysis of epithelial cells. Hyphal forms of candidiasis are seen on black arrows.

**Figure 2:**
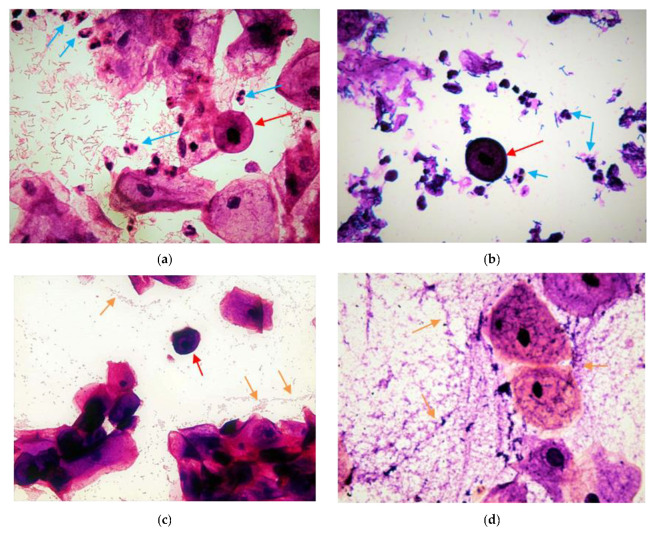
Phase-contrast images in 400× magnification after Gram staining. (**a**) AV with lactobacilli grade I. (**b**) AV with lactobacilli grade IIb. (**c**) AV with lactobacilli grade III (A microflora devoid of lactobacillus morphotypes). (**d**) Phase-contrast images in 1000× magnification full bacterial vaginosis; the red arrows illustrate the parabasal cells sign of vaginitis; the blue arrows indicate leukocytes; the orange arrows indicate the biofilm of coccoid bacteria.

To differentiate AV from other vaginal flora disorders, such as BV or candidiasis, microscopy is used (Figure 1d,e and Figure 2d) [17,18,28]. Swabs were immediately smeared on slides, air-dried, and then stained according to Gram’s method [29]. To evaluate disturbances of the vaginal secretion, the smears were then examined under a microscope (Boeco; Hamburg, Germany) (magnifications 400× and 1000×) in order to check for lactobacillus grades (Figure 2a–c), presence of clue cells, basal epithelial cells, and cocci (Figure 2). In the current study we focused on AV, and smears were analyzed using Donders’ classification method and Dong’s modified AV diagnosis for Gram stains. [17,30] ‘Any AV’ was defined as an AV score of 4 or more, with a subclassification of ‘light AV’ if the score was 4–5, ‘moderate AV’ if it was between 6–7, and ‘severe AV’ when it was 8–10.

Donders et al. 2002 characterized LBG as follows. Five criteria were studied and scored. First, lactobacillus grades (LBG) were decided; second, numbers of parabasal cells (PBC) were counted in relation to the number of human epithelial cells (EC, proportional percentage of PBC/EC). PBC were important markers of intense inflammation and mucosal thinning and/or ulceration. This sign is not seen in uncomplicated BV, but occurs frequently in severe forms of AV, and sometimes in moderate AV. Thirdly, the number of leukocytes was compared with the number of epitheliocytes, as the former are a sign of inflammatory vaginitis. Fourthly, toxic leukocytes: some leukocytes demonstrate high metabolic activity and appear in wet mount microscopy as swollen, rounded cells filled with lysosymes, coined ‘toxic leukocytes’. Finally, background microflora were studied (Table 1). In this study, the number of leukocytes and the proportion of PBC were evaluated by phase contrast microscopy (400× magnification), while LBG and background flora were evaluated by oil immersion on Gram-stained specimens (1000× magnification) (Figure 2). Clinical symptoms, including vaginal discharge consistency and color, dyspareunia, and itching, were registered for all patients (Table 1).

### 2.5. Bacterial Strains and Culture Conditions

Standard microbiological procedures were used to culture vaginal discharge samples obtained using sterile cotton swabs. [17,18,31,32].

Based on classical culture methods, cervicovaginal specimens were inoculated for bacteria isolation on mannitol salt agar (MSA) (Oxoid, Hampshire, UK), eosin methylene blue agar (EMB) (Bio-Rad, Paris, France), 5% blood agar (BA) (Oxoid, UK), and Man, Rogosa and Sharpe agar (*MRS*) (Biokar, Allonne, France). Incubation was performed for 18–24 h at 37 °C under aerobic conditions for MSA to isolate staphylococcus strains and EMB for enterobacterales strains. Additionally, the anaerobic condition was used with MRS for lactobacilli strains and BA to isolate other pathogenic bacteria, such as streptococcus and enterococcus.

We selected 50 bacterial strains for sequencing and molecular identification. From each woman with AV (42/65), the most dominant bacterial strain was used for molecular identification, while in two cases, two equally dominating bacterial strains were selected, with a resulting total of 44 pathogenic strains. In addition, six lactobacillus strains were isolated from the control women. These 50 colonies were retained, purified, and then identified by conventional phenotypic, biochemical, and molecular methods. Single strains of bacterial pathogens were initially identified using colony-shaped Gram stain and hemolytic activity. The purification was carried out with four successive subcultures spreading in solid medium specific for each species already mentioned.

#### 2.5.1. Molecular Identification of Cultivated Isolates

The different strain isolates were genotypically identified based on the 16S rRNA gene sequencing analysis. Total bacterial genomic DNA of different selected strains was extracted following the manufacturer’s instructions protocols with an All-In-One DNA Miniprep Kit (BIO-BASIC, Markham, ON, Canada). DNA concentration and purity were determined using the NanoDrop 2000, (Thermo Fisher Scientific, Waltham, MA, USA). DNA integrity and size were checked by 1.0% agarose gel electrophoresis. The V3-V4 region of the bacterial 16S rRNA gene was amplified by PCR using primers 27F/1492R [33]. PCR reactions were carried out in a final volume of 50 μL, containing 5 µL 10× PCR-Buffer (Thermo Fisher Scientific, Waltham, MA, USA), 120 µM of each dNTP (10 mM) (Jena-Bioscience, Jena, Germany), 50 pmol of each primer (10 µM) (BIO-BASIC, Markham, ON, Canada), about 50 ng of template DNA, and 2.5 U of Taq polymerase (Paq5000, 5 U/µL) (Thermo Fisher Scientific, Waltham, MA, USA ).

The cycling program was followed with denaturation at 94 °C for 5 min, 35 cycles of 94 °C, 30 s, 55 °C, 30 s, and 72 °C for 90 s, and a final extension was performed at 72 °C for 10 min (Thermal Cycler; MultiGene™, Labnet, Edison, NJ, USA). The PCR products were resolved by electrophoresis using a 2.0% agarose gel in 1X TBE buffer (89 mM Tris–borate, 89 mM boric acid, 2 mM EDTA; pH 8.0), and visualized with Gel documentation (Syngene TM, Cambridge, UK).

#### 2.5.2. Molecular Profiling and Sequencing Analysis

Amplification was confirmed by gel electrophoresis and amplicons were cleaned with the DNA Clean Kit and submitted for Sanger sequencing (Ran Biolinks, UK). Sequence alignments were performed using BioEdit software (version 7.1.11). The 16S rRNA sequences were compared to those available in GenBank DNA databases and the standard nucleotide–nucleotide BLAST algorithm for taxonomic identification of each cultivated isolate. The identities of the sequences were determined on the basis of the highest percentage of total nucleotide match in GenBank. Phylogenetic tree were constructed using MEGA software (version 6). Select strains 16S rRNA sequences were submitted to the National Center for Biotechnology Information GenBank database and the accession numbers assigned to vaginal strains were obtained [34].

#### 2.5.3. Pathogenic Properties Assays

- Acid-resistance assays

The acid tolerance of the isolated pathogenic bacteria was tested as described previously, with minor modifications [35]. In order to analyze the growth of bacteria under different pH values, pathogenic isolates were inoculated in BHI medium (Difco, France) adjusted to pH 3.5, 4, and 4.5 with 1 M HCl and 1 M NaOH. Cultures inoculated in non-acidified BHI (pH 7.0) were used as controls to compare the growth of bacteria under normal and acidic conditions. Additionally, to compare with reference bacteria growth, *Enterococcus faecalis* ATCC 29212, *Staphylococcus aureus* ATCC 25923, *E. coli* ATCC 35218, and *Streptococcus mutans* ATCC 25,175 (Kwik-stik™) were used as standards. Viable counts were evaluated initially at time zero and at timed intervals thereafter by acid challenge at 6 h, 12 h, 18 h, and 24 h growth at 37 °C in BHI. The results were expressed as the log of colony forming units per milliliter (log cfu/mL). All survival experiments were performed at least three times.

- Antibiotics Susceptibility Test

The antibiotic susceptibility of the isolates was tested using the Kirby–Bauer disc diffusion method with Mueller Hinton MH agar (BD-Difco, Strasbourg, France) and 5% sheep blood agar for the exigent strains. Thereafter, resistance data were interpreted according to EUCAST-2020 guidelines. The antimicrobial agents were tetracycline (30 μg), ampicillin (2 μg) ciprofloxacin (5 μg), gentamycin (10 μg), high-level gentamicin (30 µg) and (500 μg), high-level streptomycin (300 µg), erythromycin (15 μg), vancomycin (5 µg), clindamycin (2 μg), oxacillin (1 μg), penicillin G (1 μg), and kanamycin (30), (Oxoid, UK) [36]. *Enterococcus faecalis* ATCC 29,212, *Staphylococcus aureus* ATCC 25,923, and *E. coli* ATCC 35,218 were used for quality control for antimicrobial susceptibility testing [12].

- Biofilm Formation Assay

The experiment was carried out in two ways. The biofilm formation of the isolates was determined qualitatively on Congo red agar and quantitatively using the microtitration plate method.

Isolated strains were cultivated on the Congo red agar (CRA) containing 1 L BHIA (BD-Difco, France) with 0.08% (*w*/*v*) Congo red (Sigma-Aldrich, Germany) supplemented with 5% (*w*/*v*) sucrose (Dinamica, SP, Brazil). The strains were seeded in streaks and incubated at 37 °C under aerobic conditions for 24 h and 48 h. On CRA, slime-producing strains form black colonies, whereas non-producing strains develop red colonies [37]. All the clinical isolates included in the study were evaluated for their capacity for biofilm production, using the standard test for detection of biofilm formation according to Stepanovic et al., [38]. A microplate reader was used to quantify the optical density OD at 570 nm (Multiskan, Thermo Scientific, Waltham, MA, USA). The results were interpreted based on the optical densities of the isolates, and the production of biofilm by various strains was categorized. Bacteria were classified as high, moderate, low, and non-biofilm producers based on their OD 570 values, which were (OD ≥ 2), (1 ≤ OD < 2), (0.5 < OD < 1), and (OD ≤ 0.5) [39].

### 2.6. Statistical Analysis

For all patients, demographic data, information about pregnancy outcomes, and genital symptoms were recorded. SPSS 24.0 software was used for data processing and statistical analysis. Both bivariate analysis and multivariate regression analysis were used to evaluate the correlation of different variables with RPL and with AV.

For bivariate analysis, the significance of differences between the two groups was evaluated using the Student T-tests and the Pearson’s chi-square χ2 test. Then we process the step-wise multivariate regression analysis of the RPL group with all variables that reveal a significant correlation using bivariate analysis significant given when *p* < 0.05. To express any significant difference, estimates with 95% confidence intervals were obtained using regression analysis. For multivariate analysis, statistically significant differences were assumed for *p* < 0.05.

## 3. Results

### 3.1. Demographic and Clinical Characteristics

The mean number of pregnancy losses in the RPL group was three, most of which were before 14 weeks (70.3%), while 29.7% were between 14 and 20 weeks. The mean age of the participants in the case group (35.5 years, range 20–48 years) was similar to that of the control population (34.3 years, range 25–42 years) (Table 2). Additionally, the mean body mass index (BMI) was similar in both groups (26.5 vs. 26.8 in case and control groups, respectively). Educational levels revealed that 27.7% of patients with RPL were illiterate vs. only 8% for control-group women (OR = 4.40; 95% confidence interval (CI_95_): 1.39–14.0, *p* < 0.05). The percentages of those who had a high-school level education were 10.8% vs. 46%, in the case and control groups, respectively (*p* < 0.05).

Patients who lived in the urban regions had RPL less frequently (36.9%) than those who lived in rural areas (63.1%), (OR = 0.18; CI_95_: 0.08–0.42, *p* < 0.05). Regarding the clinical features, women with a history of RPL suffered more frequently from irregular menstruation than control-group women (80.0% vs. 12.3%) (OR = 28.5; CI_95_: 10.34–78.54, *p* < 0.0001). According to our bivariate analyses, this disorder of menstrual irregularity was also considerably correlated with the presence of AV in the study women (OR = 11.76; CI_95_: 4.38–31.60, *p* < 0.0001); this was also validated by multivariate analyses (OR = 11.02; CI_95_: 4.03–30.08, *p* < 0.0001).

Mean vaginal pH, an indirect indicator of vaginal dysbiosis, was higher in RPL women (pH 4.9, range 3.8–6.3) than in the control group (pH 4.2, range 3.8–4.9), *p* < 0.0001) (Table 2). In addition to abnormal pH levels, we found 58.5% of women in the RPL group to have vulvovaginal symptoms vs. 8% in the control group (OR = 0.06; CI_95_: 0.02–0.19, *p* < 0.0001).

### 3.2. Prevalence of Aerobic Vaginitis in Study Population

Results showed that the prevalence of all AV (composite AV score ≥ zero, Table 1) was significantly higher in patients who had previous recurrent pregnancy loss (70.7%) than in those without (12%) (OR = 16.21; CI95: 5.90–44.53, *p* < 0.0001) (Table 2). Moderate–severe AV (AV score > four) was 50 times more frequent in the group with an RPL history compared to the controls (OR 50.63, CI_95_: 6.57–390.39), *p* <0.0001). Twenty-nine percent of RPL women had normal vaginal flora on microscopy (Figure 1a), while 15.3% suffered from severe AV, 32.3% had moderate AV, and 16.9% had mild AV, compared to 0%, 2%, and 10%, respectively, in the normal group (*p* < 0.0001) (Figure 1b,c and Figure 2). Other genital infections, such as candidiasis and BV, were found in 6.2% of the study participants and not in the control-group women (n.s) (Figure 1d,e and Table 2).

The multivariate analysis regarding variables that revealed a significant bivariate association with RPL indicated that menstrual irregularity and AV are strongly related to RPL (OR = 21.15; 95%, CI_95_: 6.05–73.91, *p* <0.0001 and OR = 7.18; CI_95_: 1.90–27.10, *p* < 0.05, respectively). However, the residence area was statically linked with a history of RPL, (OR = 0.18; CI_95_: 0.05–0.64, *p* < 0.05), (Table 3 and Table S1).

### 3.3. Microbial Diversity and Molecular Analysis

**Figure 3:**
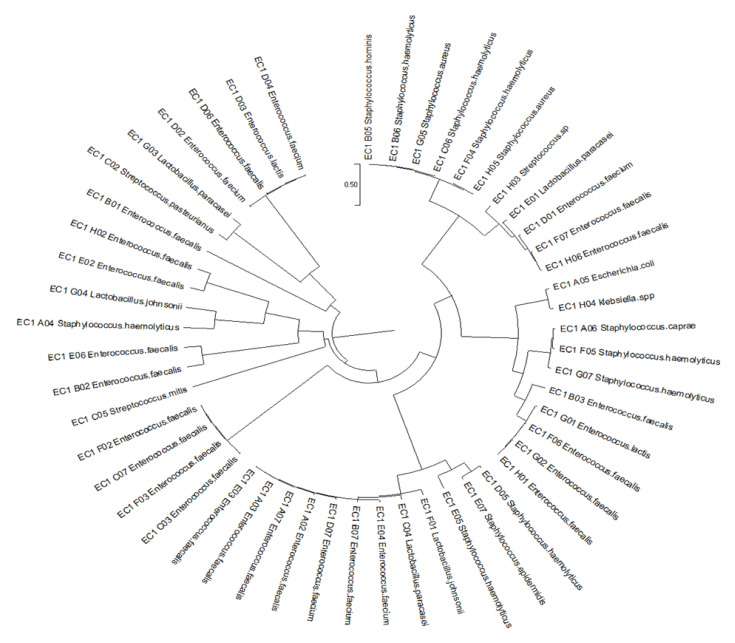
Phylogenetic tree of the isolated strains using UPGMA methods.

A total of 50 strains were chosen to be identified by molecular method, of which 96% were Gram positive and 4% were Gram negative. Of all strains, 65.4% were hemolytic, whereas 34.6% were non-hemolytic. The sequencing findings suggest that the most prevalent isolates were *Enterococcus* sp. (52.0%), *Staphylococcus* sp. (26.0%), *Streptococcus* sp. (6.0%), and sporadic *E. coli*, *Klebsiella* spp., and six lactobacilli strains (Table 4). Next, a phylogenetic tree was constructed using all the identified strains (Figure 3).

### 3.4. Acid Susceptibility Test

**Figure 4:**
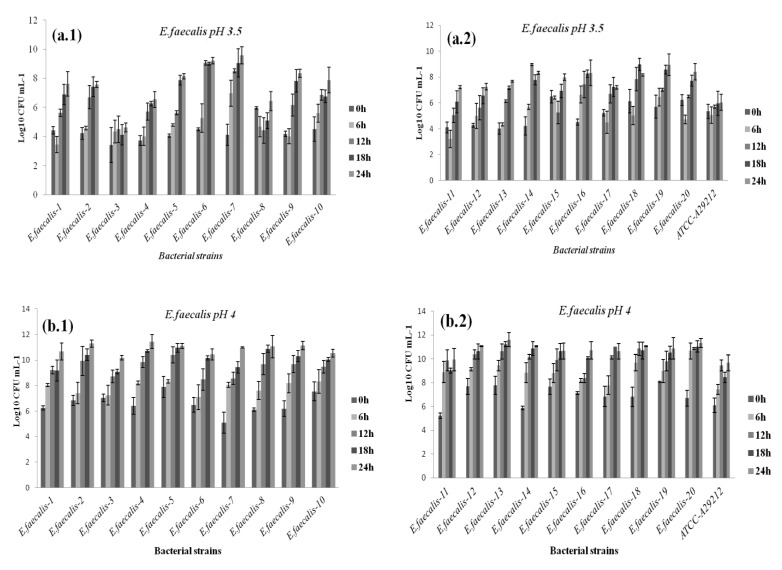

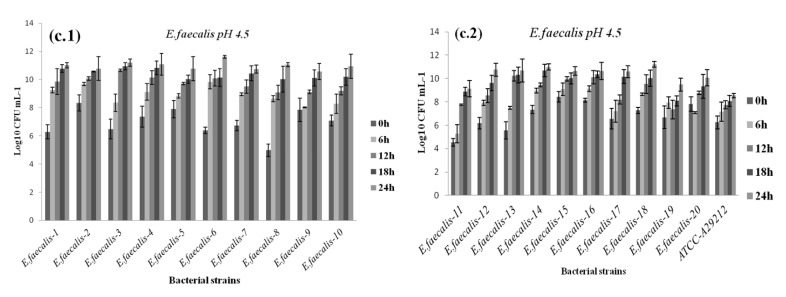
Growth of vaginal *Enterococcus faecalis* strains at pH 3.5 (**a1**,**a2**); pH 4 (**b1**,**b2**); and pH 4.5 (**c1**,**c2**).

**Figure 5:**
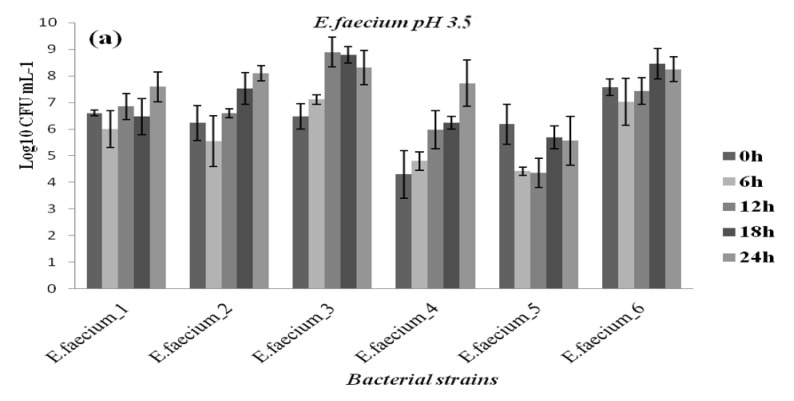

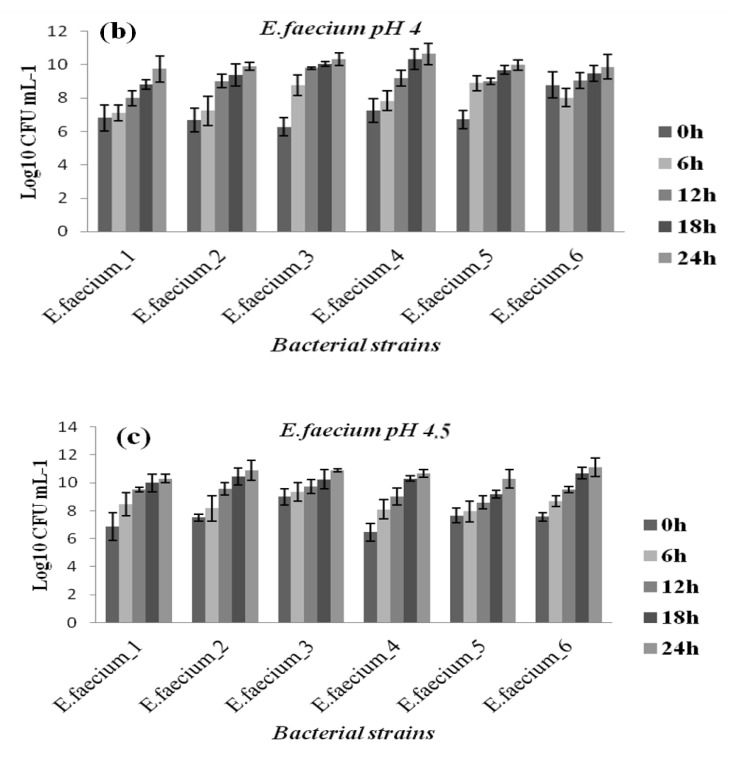
Growth of vaginal *Enterococcus faecium* strains at pH 3.5 (**a**); pH 4 (**b**); and pH 4.5 (**c**).

**Figure 6:**
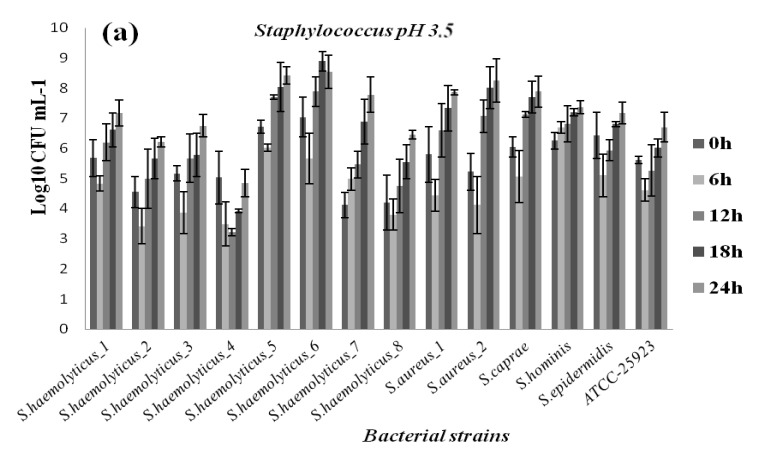

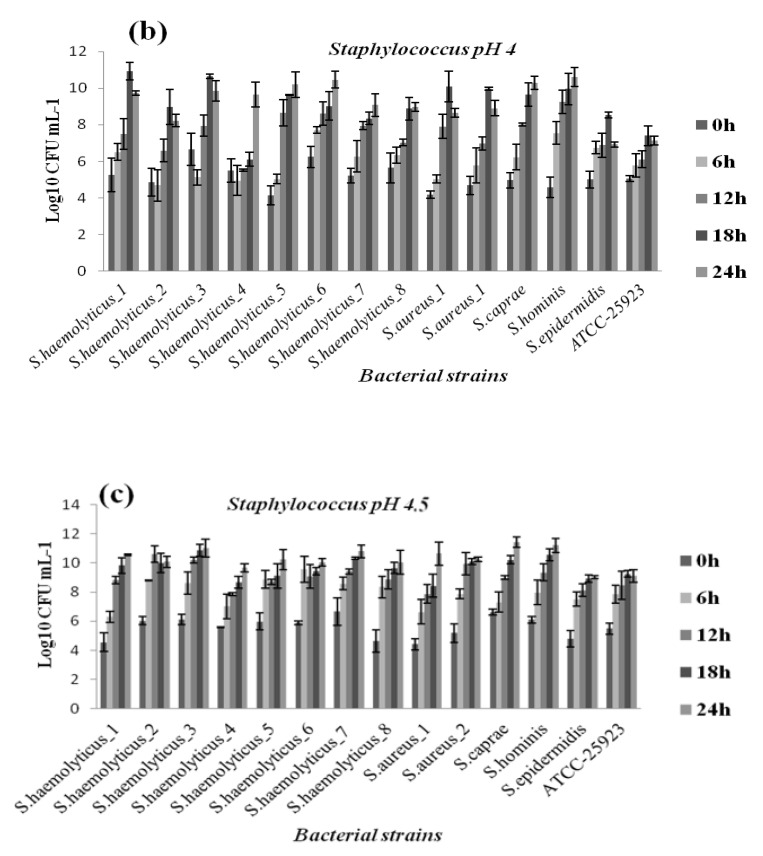
Growth of vaginal staphylococcus strains at pH 3.5 (**a**); pH 4 (**b**); and pH 4.5 (**c**).

**Figure 7:**
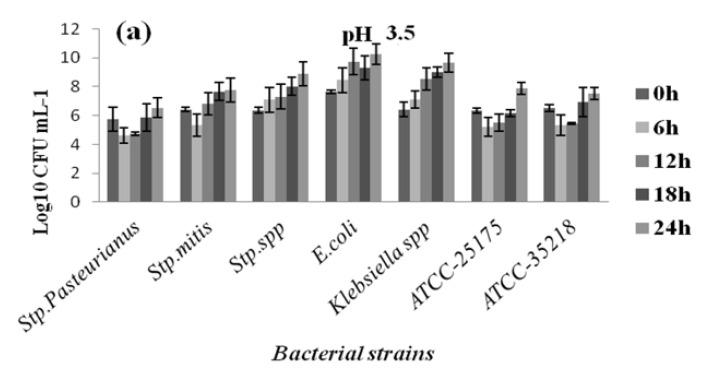

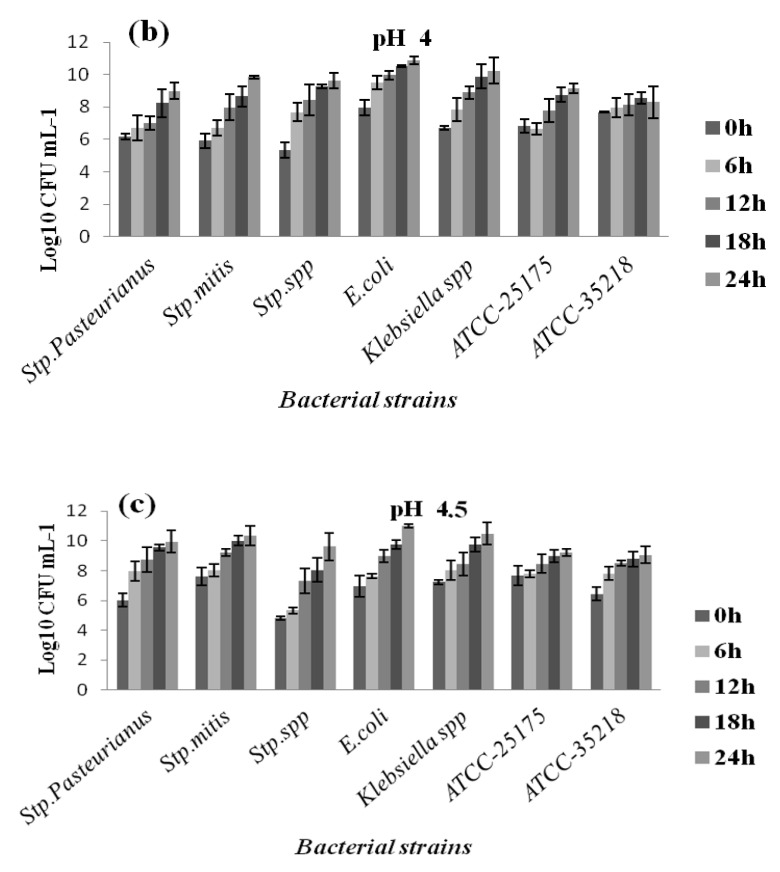
Growth of vaginal streptococcus, *E. coli,* and *Klebsiella* spp. strains at pH 3.5 (**a**); pH 4 (**b**); and pH 4.5 (**c**).

At all pH levels and when compared to reference strains, better growth potential was demonstrated by the pathogenic isolates. Furthermore, all bacteria strains showed reduced growth at pH 3.5, 4, and 4.5 compared to pH 7, although they appeared to tolerate low pH well, albeit at different levels. The results are reported in Figure 4, Figure 5, Figure 6 and Figure 7 in the appendix. Based on the results, even after 24 h of exposure to different pH levels, viability was retained by the 44 selected strains. Comparing the behaviors of different bacterial groups evaluated in this study at varied pH values and based on survival rates, it was notable that *E. faecalis* usually presented the highest survival rate independent of acid stress conditions (Figure 4).

In contrast, the genus staphylococci seemed to be more vulnerable to acid conditions based on their weak survival rate at pH 3.5 (Figure 6a). Bacterial multiplication revealed by the viable cell count (log cfu/mL) showed that staphylococci were the most susceptible genus at pH 3.5, as opposed to *E. faecalis*, which had the highest growth rate, followed by streptococcus and *E. faecium* (Figure 5). Compared to pH 3.5, all strains showed improved growth at pH 4. In addition, we noted that the strain’s multiplication improved and strains grew successfully at pH 4.5. As a result, the maximum bacteria number in pH 4.5 after 24 h ranged from 11.62 log cfu/mL in *E. faecalis* to 9.01 log cfu/mL in *Staphylococcus epidermidis*. The result suggests that *enterococci* are much more tolerant to acidic stress than staphylococci and streptococci (Figure 7). The survival rate of the streptococcus strains increased with increasing pH; thus, the 24 h viability improved with increasing pH levels. The number of viable *Streptococcus* spp. strains after 24 h ranged from 8.87 log cfu/mL to 10.33 log cfu/mL at pH 3.5 and 4.5, respectively. The enterobacterales species (*Escherichia coli, Klebsiella* spp.) from the patients were found to be tolerant under varied acid stress conditions, indicating they had good survival when compared to bacterial multiplication at pH 7 and compared to *E. coli* ATCC 35,218 survival rates (Figure 7). It was evident that, at all acidic conditions, strains reacted differently; this suggests that their survival ability is a strain-specific property.

### 3.5. Drug Susceptibility Profiles

Drug sensitivity tests were carried out only for the 44 pathogenic strains, excluding the six lactobacillary strains (Table 5). Tetracycline, quinolone, aminoglycosides, and glycopeptides were the families with the greatest efficacy towards most tested strains. The prevalence of multidrug resistance among all bacteria was 47.7% (21/44).

Results showed that the least active of the drugs evaluated on all Gram-positive bacteria was erythromycin (macrolides) at a resistance of 83.3%, followed by tetracycline 73.8%, aminoglycosides 66.7% (gentamicin, kanamycin), ciprofloxacin 62.5%, vancomycin 59.5%, and clindamycin 56.2%. The lowest resistance rate was in beta-lactamines, but it was still significant at 50.8% (oxacillin, PG, ampicillin). The tested strains developed 72.4% and 51.7% antimicrobial resistance to high levels (HL) of streptomycin and gentamicin, respectively. For *Enterococci* (*faecalis* and *faecium*), the most frequent genus, resistance was detected toward five antibiotic families. In addition to their multidrug tolerance, both species showed resistance to gentamicin and streptomycin at high levels. They exhibited resistance rates ranging from 45–100% to high-level aminoglycosides tested. For Gram-positive bacteria, *E. faecalis* was found to be less tolerant than *E. faecium*. Indeed, most of the *E. faecium* strains were seen to be more tolerant to ampicillin (66.7%) than *E. faecalis* (35%). The maximum resistance of *E. faecalis* was recorded for vancomycin at 95%.

For the second commonly isolated species, *S. haemolyticus*, a high rate of resistance was noticed against tetracycline, kanamycin (50%), ciprofloxacin, gentamicin (62.5%), erythromycin, and clindamycin (75%). The overall drug sensitivity profile of *S. aureus* revealed resistance to oxacillin, gentamicin, kanamycin, clindamycin, and ciprofloxacin among both strains, while *S. caprae*, *S. hominis*, and *S. epidermidis* reported sensibility to kanamycin, clindamycin, and vancomycin. The streptococcus strains’ resistance was quite significant against the tested antibiotics. Indeed, tetracycline, erythromycin, and streptomycin at high concentrations had no effect on this bacteria genus. In addition, two of three tested streptococcus were tolerant to a high concentration of gentamycin. However, for streptococcus strains, no resistance to vancomycin was shown. The current data reported that the tested enterobacterales were susceptible to kanamycin. However, they indicated total resistance to gentamycin and ciprofloxacin. Resistance patterns for all strains are reported in Table 5.

### 3.6. Qualitative and Quantitative Ability for Biofilm Formation

**Figure 8:**
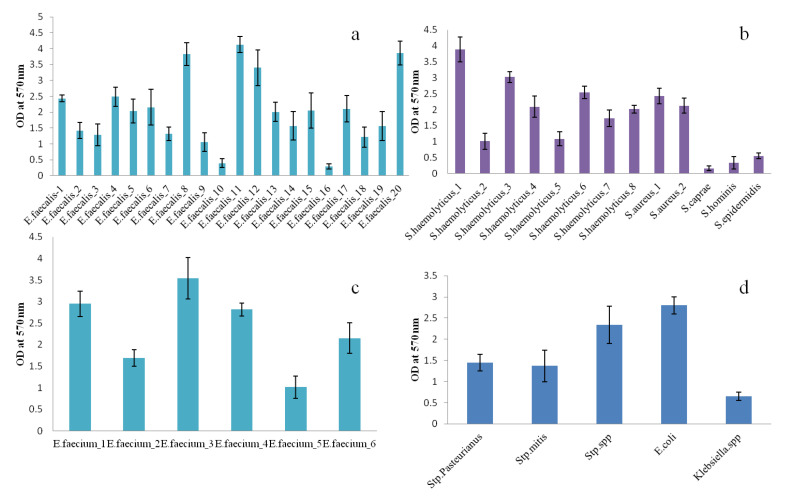
The biofilm formation by the different strains quantified by crystal violet staining. (**a**) *Enterococcus faecalis* strains; (**b**) staphylococcus strain; (**c**) *Enterococcus faecium* strains; (**d**) Stp: Streptococcus strains and enterobacterales strains.

**Figure 9:**
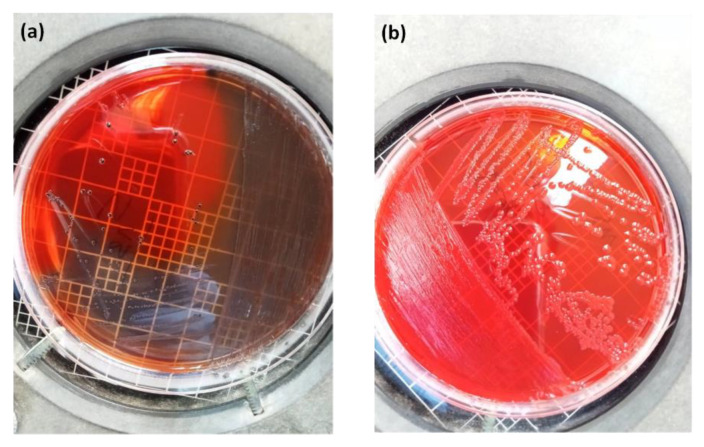
The appearance of strains cultured on Congo red agar. (**a**) colonies of *Enterococcus faecalis* Slime-producer; (**b**) *Staphylococcus hominis* strain, non-producing slime.

**Figure 10:**
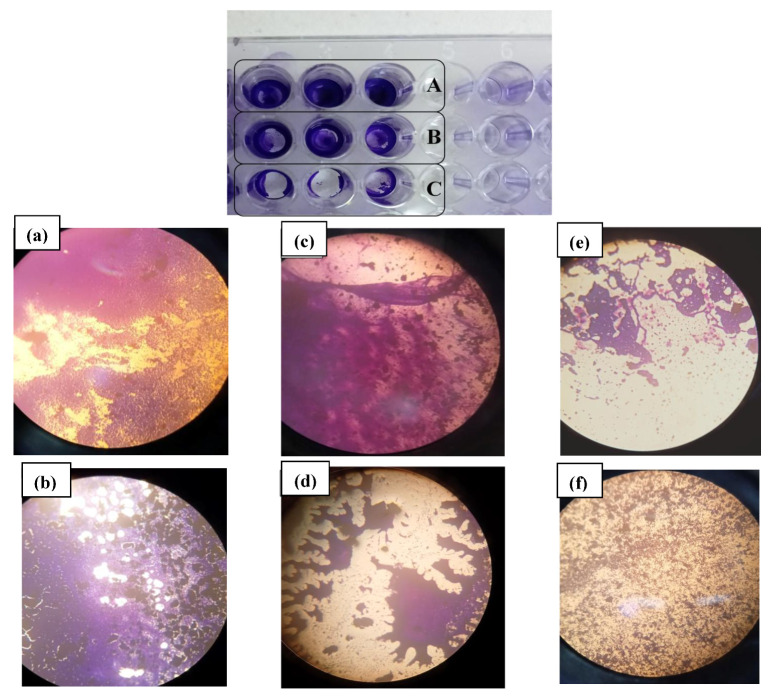
Bacterial biofilm formation on the surface of polystyrene. (**A**) *E. faecalis* strain appears to be strong biofilm producer; (**B**) *E. faecalis* strain appears to be moderate biofilm producer; (**C**) *E. faecalis* strain appears to be a weak biofilm producer. Phase-contrast images in 400× magnification showing the development of biofilm bacteria on a polystyrene plate; (**a**)–(**c**) *E. faecalis* strain with a high biofilm formation; (**d**,**e**) *E. faecalis* strains with a moderate biofilm formation, and (**f**) *E. faecalis* strain presents a low biofilm formation.

Ninety-one percent of the 44 strains were detected as quantitative biofilm producers, and among them, more than 54% (24/44) were high biofilm producers, 31% (14/44) were moderate producers, and 4% had low biofilm formation potential (Figure 8). A strong rate of biofilm formation of 92.3% (24/26) was revealed by the *Enterococci* genus. Furthermore, 80% of them were slime producers on the Congo red agar (Figure 9). Additionally, a high potential of biofilm production was expressed by more than 50% of staphylococci, and the majority of them were slime producers. In particular, *S. aureus* and the majority of the *S. haemolyticus* strains appeared to be strong biofilm producers. With the two methods, no biofilm creation was observed in *S. caprae* and *S. hominis*. All streptococcus strains appeared able to produce biofilm, as analyzed using the qualitative and quantitative methods. The *E. coli* and *Klebsiella* spp. strains were strong and weak biofilm producers, respectively (Figure 10).

## 4. Discussion

According to some authors, pregnant women may be more susceptible to vulvovaginal infections than non-pregnant women [40], and the severity of vaginal infection may increase during pregnancy [41]. AV is an endogenous opportunistic infection that is associated with various complications during pregnancy, such as fetal infections, preterm birth, and miscarriage [16,21,42,43,44]. Besides the multiple known potential causes of RPL, vaginal microbial imbalance is also associated with RM, according to several recent studies [45,46,47,48].

The overall prevalence of AV in the control population of our study was 12%, which is comparable to the rate of moderate/severe AV in 11% of women presenting for a routine gynecological check-in in Uganda [49] and in Bulgaria [50]. Our AV prevalence is also consistent with previous reports of asymptomatic normal pregnant women, which was estimated to be between 7% and 13% in numerous studies in Europe, Asia, and Africa [14]. In contrast with this, the prevalence of AV in our study women with a history of RPL was five-fold higher (64.6%). These findings are comparable to data from others who observed that AV is the major vaginal disorder in symptomatic non-pregnant women, with a rate of 51% [51,52]. The AV prevalence in women with a reproductive history of RPL appears to be comparable with the data of Cicinelli et al., where common bacteria, including AV-causing bacteria, affected almost 60% of women with RPL in Italy [53].

The strong correlation of AV with RPL in our study suggests AV has a possible role in the etiology of RPL. This corresponds with the hypothesis that AV may be a more important cause of pregnancy complications than BV [18]. In agreement with this, an imbalance of the vaginal ecosystem due to AV bacteria was also suggested by other data to represent a significant contribution to the development of recurrent miscarriages [22,48,54].

Ascending infection generated by vaginal microorganisms can lead to a better understanding of how AV induces pregnancy complications [43]. An inflammatory genital tract reaction was mediated by AV bacteria, and a host immune response was generally induced by AV infection [55]. Following the ascending infection, or even from inflammatory responses in the vagina itself, the uterine inflammatory response created by AV bacteria and/or their metabolites could have a serious impact on the pregnancy, including intrauterine infection and miscarriage [8,55].

In this study, we also identified that 41.5% of AV cases were diagnosed in asymptomatic women. This is comparable to other research reported by Salinas et al., where 49% of AV was detected in asymptomatic women. In support of these findings, there is clear evidence that, even when asymptomatic, AV is a potential cause of pregnancy complications, particularly fetal infection [56]. Hence, we agree with Kaambo et al. that screening for vaginal and cervical microflora aberrations needs to be considered by clinicians while exploring the diagnosis RPL [57].

Indeed, even after multivariate analysis, AV remained a main independent risk factor for a history of RPL, alongside some other, well-known factors indicating lower socio-economic status. In this study, there was no difference in the mean age of women with RPL and controls, indicating age was not a risk factor for recurrent miscarriage. However, maternal age above 35 was associated with an increased risk of fetal loss in other studies [58].

The bivariate analysis finding that lower education was associated with RPL, confirmed by other authors [59], but after multivariate analysis, this factor was no longer significant in our series. On the contrary, living in rural areas was a significant risk factor for increased risk of RPL, both after bivariate and multivariate analysis, which was confirmed by others [59,60].

The vaginal pH levels in reproductive-age women without vulvovaginal symptoms vary between 3.8 and 4.4 [61]. Elevated vaginal pH, that is, above 4.7, or 4.5 according to others, is recognized to be the most significant indicator of abnormal conditions in the vaginal ecosystem, such as BV or AV [18,49]. Higher pH values in women with RPL compared to women in the control group were confirmed by our data. According to Fan et al., 87.5% of AV patients have a vaginal pH of 4.5 or higher [13].

A remarkable clinical observation confirmed also by multivariate analysis was that irregular menstrual cycles were much more common in women with a RPL history. Strikingly, in Sugiura-Ogasawara’s study, a history of irregular menstrual periods was associated with recurrent miscarriages [62]. Therefore, we hypothesize that the composition of the vaginal microbiota is affected by hormonal fluctuation and longer periods of menstrual blood loss [63]. Our data confirmed that the presence of irregular menstruation is indeed clearly associated with AV, and this association remained strong after multivariate analysis. It is known that the diversity and abundance of vaginal microbiota are often influenced by the time of the menstrual cycle [64]. *Lactobacillus* species have been reported to be the most negatively affected by menstruation [65].

In order to understand the virulence factors of our isolated pathogenic bacteria, we started by testing their ability to survive under different vaginal pH conditions. Our findings suggest that tested isolates are highly adapted to the vaginal specialized niche: the majority of isolates were able to survive and multiply under acidic conditions. Clinical evidence supports that colonization with lactobacillus species, resulting in a low pH of 3.5–4.5, has been shown to protect women against vaginal dysbiosis and adverse pregnancy outcomes [66]. Of note, the tested bacterial strains appeared to tolerate low pH in different ways, because they used different mechanisms of tolerance. Based on these findings, enterococcus and streptococcus acid resistance can be explained by one of the major acid stress response mechanisms in both species, F0F1 ATPase regulation, as reported by Zhou et al. [66]. The same study explains the staphylococcus genus acid resistance pathway by the Arginine Deiminase resistance mechanism, which is conserved across many staphylococcal species, including *S. epidermidis*, *S. aureus*, and *S. haemolyticus* [67]. This resistance is crucial, given that acidification is often considered as a therapy strategy against vaginal infection, because most pathogens fail to grow at a pH below 4.5, and this acidic environment facilitates the growth of protective lactobacillus species [68].

Therapy failure and continued administration of antimicrobial drugs to manage vaginitis could result in the emergence of antibiotic resistance [57]. In our study, a multidrug-resistant (MDR) organism was observed in 47.7% of all tested isolates. This was similar to the MDR rate of 50.9% found by others [69], and can be explained by biofilm formation, given that 91% of our strains were biofilm producers. In results comparable to our findings, Farinati et al. observed biofilm formation by similar bacterial genera in women with vaginal infections [70]. In our series, the overall drug sensitivity profiles of Gram-positive bacterial isolates varied from the most potent antibiotic, beta-lactamine, to the less effective erythromycin. Similar research showed that the highest tolerance of Gram-positive bacteria was found with erythromycin, at 75.8% [71]. Except for kanamycin, Gram-negative bacteria identified appeared resistant to all the antibiotics tested. Sometimes, drugs such as kanamycin, tetracycline, and quinolones are prescribed to treat for vaginitis. [28,72]. The eradication of the tested enterobacterales species was only obtained by the use of kanamycin. This was comparable to the conclusions of Tempera et al., who reported significant antimicrobial effects with topical kanamycin (97%) in AV associated with enterobacterales [73]. The significant resistance level expressed by *E. faecalis* and *E. faecium* toward the aminoglycosides assessed was not surprising, given the fact that enterococci are considered to be hard to eradicate because of their intrinsic resistance to antibiotics, particularly aminoglycosides. Certainly, there is a significant increase in the occurrence and propagation of high-level resistance (HLR) to aminoglycosides, which have historically been the main active anti-enterococcal antibiotics [74]. Our findings are in accordance with the results of Serretiello et al., who identified a significant rate of resistance to high-concentration gentamicin and streptomycin. On the other hand, alarming resistance to vancomycin was recorded for our identified Enterococci. Furthermore, an important vancomycin-resistant enterococcus was observed in our isolated *E. faecalis* (95%) and *E. faecium* (50%), which at the same time showed high biofilm-forming ability. This was correlated with the recent suggestion of an increase in the emergence of vancomycin-resistant *Enterococci* [75]. The ability of vaginitis-related enterococcus to produce biofilm has been reported by many studies as an important virulence factor. [39]. We hypothesize that chronically persistent biofilms of Enterococci in the genital tract can explain recurrent AV episodes, thereby contributing to pregnancy complications, such as RPL.

As a result, trying to find an antibiotic for germs is not the only way, and probably not the best way to protect women against AV and pregnancy outcomes. More importantly, useless, excessive application of antibiotics for treating vaginal culture results may inevitably result in this alarmingly high MDR. As a consequence, this study supports and highlights the importance of an alternative therapy based on probiotics or immune modulators (like local estrogen or progesterone) as an approach to decreasing AV and its consequences, rather than using ever more developed antibiotics that may amplify the problem of MDR and not solve this issue. Amid the global alarm over antibiotic resistance, researchers are investigating alternative ways that have fewer adverse effects and do not damage the natural microflora. Indeed, studies suggest that management of AV would need to be a multifactorial approach rather than a single antibiotic treatment, which includes probiotics and hormonal enhancements [28,76]. Han et al. similarly advised that the recommended strategy for treating AV should be based on microscopy evidence, a local therapy with required antibiotics to target the causative agent, as well as topical steroids to decrease inflammation and estrogen to manage the atrophy [31]. Since hormonal modulation is crucial for pregnancy control, local administration of estradiol or estriol, single or combined with probiotic lactobacilli, may be considered in cases where AV-related atrophy has resulted in higher numbers of parabasal cells. This is not possible in all patient cases, particularly in women with contraindications to steroid hormone therapy, which includes breast cancer [76]. However, probiotics, particularly lactobacillus species, have shown considerable promise as a possible therapy [77]. Recently, a combined intravaginal probiotic and low-dose estriol was reported to be successful in avoiding the occurrence of AV [78]. The application of exogenous lactobacilli can provide a considerably safer alternative pathway to prevent MDR and AV-related atrophy recurrence, and restore defensive vaginal flora [31,79]. The contribution of lactobacilli to an optimal genital microbiota is extremely important to maintaining pregnancy. These probiotics are also promoted to retain the equilibrium of vaginal homeostasis and immune regulation by expressing properties that are antagonistic to pathogens, while being complementary to host immunity [77] and leading to increased conception rates and decreased vaginal infection risk, as well as reducing the risk of a variety of pregnancy disorders. Many studies report that specific single or mixed strains of probiotics, alone or in combination with standard drug treatments, either orally or vaginally, have demonstrated great potential as an alternative strategy to improve the vaginal flora and thus minimize the number of harmful bacteria. Even in the absence of clinical symptoms of vaginitis, administration of probiotics was helpful in establishing a healthy ecosystem and reducing the recurrence of vaginal infection, thus preventing pregnancy complications and adverse outcomes [80,81,82,83].

In normal circumstances, lactobacillus species compete to bind to receptors on host epithelial cells, thereby inhibiting adhesion of pathogens or displacing pre-attached pathogens [84]. Biofilm formation by pathogens may limit this probiotic defense by interfering with the probiotics’ adherence to vaginal epithelial cells [85]. Dehpahni et al. studied the effect of a silver nanoparticle on bacterial biofilms and revealed that such substances are able to remove planktonic pathogen organisms, resulting in a strong anti-biofilm effect that can potentially reduce *E. coli* and *S. aureus* biofilms in the case of AV [86].

Both Caspar et al. and Cirkovic et al. independently demonstrated a novel bacteriocin being produced in probiotics that has the potential to prevent biofilm development and eradicate biofilms that are already formed, thus reducing genital and neonatal staphylococcus infections such as *S. epidermidis*, *S. hominis*, and *S. haemolyticus* [87,88]. Considering the significant biofilm formed by vancomycin-resistant enterococci (VRE) in our study, we suggest the use of lactobacilli to manage such infections. Tytgat et al. provide a molecular basis for a model for a novel probiotic mechanism using L. rhamnosus for prevention and treatment VRE infections, since this bacterium may prevent the binding of a potential pathogen to the host, thus inhibiting adhesion and biofilm formation [89]. Although in small preliminary studies, probiotics seemed effective in diminishing AV [14,16,28], large, controlled studies highlighting the value of probiotics as adjuvant therapy targeting the AV microbiota are mandatory to achieve the goal of restoring a balanced *lactobacillus*-dominated microflora in the vaginal niche [26,31,78].

In summary, AV is associated with irregular menstruation and social factors such as living in a rural area, and most importantly, AV appears a strong and independent risk factor for recurrent pregnancy loss. Given the high resistance rates and biofilm-producing abilities of all commensal and pathogenic bacteria we found associated with AV in women with a history of RPL, just providing more antibiotics is unlikely to achieve an improvement in the vaginal microflora that is significant and long-lasting enough to prevent future pregnancy losses. Furthermore, the presence of menstrual irregularities in relation to RPL history suggests that an altered hormonal milieu may contribute to pregnancy complications by inducing different vaginal microflora, such as AV. Indeed, underlying hormonal disturbances, local immune deficiencies, or estrogen receptor problems in the vagina can play a much more crucial role than the bacterial environment in the pathogenesis of AV, which leads to the theory that the microorganisms found in AV can rather be seen as a consequence rather than a cause of it [15]. Therefore, in the management of AV, other therapeutic actions than antibiotic use are central [72].

- Strong points

The current study represents a thorough research endeavor, given the large sample size, as RPL is a pregnancy complication that has been rarely studied, especially with regard to unexplained cases. Another strong aspect of the study is the detailed illustration of the AV-associated bacteria after a rigorous application of the AV diagnosis criteria. This study gives a clear global insight following the genome sequencing of these implicated bacteria from different species, and offers a deep investigation of significant pathogenesis factors, such as acid resistance and multidrug resistance, in addition to intensive biofilm-formation testing. To elaborate, the study unveils the risk factor of the presence of potentially pathogenic AV bacteria in the case of RPL.

- Shortcomings

Although the current work provides valuable information about the studied pathology, it remains an unfinished work that hopefully will be the basis for future research. However, it is not without certain shortcomings in terms of uncultured bacteria that were not included in this study. Furthermore, metagenomic sequencing of total DNA extracted may give a clearer idea about unbalanced flora than is possible with bacteria that need to be cultured for the study of pathogenic factors. One of the major challenges during this study was to avoid the risk to healthy control-group women without signs of vulvovaginitis or other vaginal disorders. Therefore, to avoid this risk, we enrolled control-group women randomly; thus, the presence of symptomatic women was not an exclusion criterion in control-group women.

## 5. Conclusions

Our results strongly support the theory that AV dysbiosis of the vaginal microflora is associated with an increased risk of RPL. Indeed, even (long) after pregnancy, the differences in menstrual abnormalities, socio-economic circumstances and the presence of msAV and increased pH remain visible and significantly more frequent in these women compared with women who had successful pregnancies. Other factors, such as age and BMI, were not related to a history of RPL. This implies that the internal dysregulation causing AV, whether this is caused by a hormonal disbalance, by an estrogen-receptor deficiency in the vagina, or by any unexplored immunological cause, is and endogenic problem that resides in the vaginal mucosa and can remain present for years, or possibly lifelong. The association of AV with pregnancy complications is thereby not necessarily one of cause-and-effect. Further studies will have to unravel the pathogenesis, but in this study we aimed to contribute to a better understanding of the characteristics of the microbiota present in AV patients by studying their acid resistance, their capacity to form biofilms, and their resistance against antibiotics. As a result of our striking finding of a five-fold increased risk of AV in women with previous pregnancy losses, we strongly recommend that clinicians should incorporate screening for AV for all women at risk of RPL, or perhaps for all women intending to become pregnant. Since AV is a complicated entity that will not always respond to common antibiotic therapies adopted for other types of vaginitis, we further recommend the consideration of broader therapeutic approaches with hormonal, anti-inflammatory, and probiotic components.

If short-term antibiotics are used, to avoid the emergence of MDR bacteria and biofilm formation, antibiotic susceptibility patterns should be taken into account. In this study, we demonstrated that pathogenic strains were able to persist in the genital tract using acid resistance to combat vaginal pH, resistance against multiple antibiotics, and the ability to produce biofilm as important pathogenic tools. We conclude that screening for such alterations in vaginal microbiota may in the long term contribute further to the understanding of the pathogenicity of AV-associated micro-organisms in order to prevent pregnancy complications. We believe this is the first in-depth investigation of the association between AV and spontaneous RPL to be reported. It calls for more investigation to elucidate the pathogenetic mechanisms and best therapeutic approaches.

## Figures and Tables

**Table 1:** Diagnostic criteria for AV Gram staining with clinical symptoms (Dong M. et al. 2022).

Score	LBG (1000×)	No of Leucocytes/Epithelial Cells (400×)	Background Flora (1000×)	Proportion of PBC (400×)	Clinical Symptoms
0	I,IIa	≤10	No other bacteria	<1%	pH ≤ 4.5 and no abnormal symptoms
1	IIb	≤10	Small bacilli	≥1% and ≤10%	pH > 4.5 or at least one abnormal symptom
2	III	>10	Cocci or chains	>10%	pH > 4.5 and at least one abnormal symptom

**Table 2:** Sociodemographic and clinical characteristics of study population and microscopic diagnosis of vaginal disorders.

Clinical Features	RPL Group	Control Group	*p*-Value
N = 115	N = 65	N = 50	*t*-Test
	Average (min-max)		
Woman’s age (years)	35.54 (20–48)	34.26 (25–42)	n.s
Body mass index (BMI) kg/m^2^	26.49 (17.19–33.79)	26.77 (19.47–34.63)	n.s
Gravidity	3.51 (2–7)	3.18 (2–5)	n.s
Parity	0.08 (0–1)	3.18 (2–5)	<0.0001 *
Vaginal Ph	4.88 (3.8–6.3)	4.24 (3.8–4.9)	<0.0001 *
	Number (%)		*p*-value *X*^2^
Level of education			<0.0001 *
No education	18 (27.7%)	4 (8.0%)	
Intermediate	40 (61.5%)	23 (46.0%)	
Higher	7 (10.8%)	23 (46.0%)	
Smoking			n.s
Yes	6 (9.2)	7 (14.0%)	
No	59 (90.8%)	43 (86.0%)	
Residence			<0.0001 *
Urban	24 (36.9%)	38 (76.0%)	
Rural	41 (63.1%)	12 (24.0%)	
Blood groups			n.s
A	17 (26.2%)	16 (32.0%)	
B	8 (12.3%)	3 (6.0%)	
O	40 (61.5%)	31 (62.0%)	
Menstrual cycle			<0.0001 *
Regular	8 (12.3%)	40 (80.0%)	
Irregular	57 (87.7%)	10 (20.0%)	
Aerobic vaginitis (AV) **			<0.0001 *
No AV	19 (29.23%)	44 (88%)	
Mild AV	11 (16.92%)	5 (10%)	
Moderate	21 (32.30%)	1 (2%)	
Severe	10 (15.38%)	0	
Bacterial vaginosis	2 (3.07%)	0	n.s
Candidosis	2 (3.07%)	0	n.s

RPL: Recurrent pregnancy loss; n.s: not significant for *p* > 0.05; * Statistically significant differences. ** AV score (<4 No AV/4–5 mild AV/6–7 moderate AV/8–10 severe AV).

**Table 3:** Multivariable regression analysis of RPL with significant factors.

Variable	Odds Ratio [OR]	95% Confidence Interval	Statistical Significance
Menstrual irregularity	29.88	9.73–91.73	<0.0001 *
Aerobic vaginitis	7.34	1.99–27.01	*p* < 0.05 *
Rural area	0.171	0.05–0.53	*p* < 0.05 *
Education level	0.226	0.04–1.05	n.s

* Statistically significant differences.

**Table 4:** Accession numbers provided by GenBank database of all strains DNA sequences.

Strain	Accession Number
*1.* *Enterococcus faecalis_1*	MZ474967
*2.* *Enterococcus faecalis_2*	MZ474968
*3.* *Enterococcus faecalis_3*	MZ474972
*4.* *Enterococcus faecalis_4*	MZ474973
*5.* *Enterococcus faecalis_5*	MZ474974
*6.* *Enterococcus faecalis_6*	MZ474975
*7.* *Enterococcus faecalis_7*	MZ474980
*8.* *Enterococcus faecalis_8*	MZ474984
*9.* *Enterococcus faecalis_9*	MZ474990
*10.* *Enterococcus faecalis_10*	MZ474993
*11.* *Enterococcus faecalis_11*	MZ474994
*12.* *Enterococcus faecalis_12*	MZ474997
*13.* *Enterococcus faecalis_13*	MZ475000
*14.* *Enterococcus faecalis_14*	MZ475001
*15.* *Enterococcus faecalis_15*	MZ475004
*16.* *Enterococcus faecalis_16*	MZ475005
*17.* *Enterococcus faecalis_17*	MZ475007
*18.* *Enterococcus faecalis_18*	MZ475012
*19.* *Enterococcus faecalis_19*	MZ475013
*20.* *Enterococcus faecalis_20*	MZ475017
*21.* *Enterococcus faecium_1*	MZ474978
*22.* *Enterococcus faecium_2*	MZ474985
*23.* *Enterococcus faecium_3*	MZ474986
*24.* *Enterococcus faecium_4*	MZ474988
*25.* *Enterococcus faecium_5*	MZ474991
*26.* *Enterococcus faecium_6*	MZ474995
*27.* *Staphylococcus haemolyticus_1*	MZ474969
*28.* *Staphylococcus haemolyticus_2*	MZ474977
*29.* *Staphylococcus haemolyticus_3*	MZ474983
*30.* *Staphylococcus haemolyticus_4*	MZ474989
*31.* *Staphylococcus haemolyticus_5*	MZ474996
*32.* *Staphylococcus haemolyticus_6*	MZ475002
*33.* *Staphylococcus haemolyticus_7*	MZ475003
*34.* *Staphylococcus haemolyticus_8*	MZ475011
*35.* *Staphylococcus aureus_1*	MZ475010
*36.* *Staphylococcus aureus_2*	MZ475016
*37.* *Staphylococcus hominis*	MZ474976
*38.* *Staphylococcus caprae*	MZ474971
*39.* *Staphylococcus. epidermidis*	MZ474998
*40.* *Streptococcus mitis*	MZ474982
*41.* *Streptococcus. pasteurianus*	MZ474979
*42.* *Streptococcus* sp.	MZ475014
*43.* *Escherichia coli*	MZ474970
*44.* *Klebsiella* spp.	MZ475015
*45.* *Lactobacillus paracasei*	MZ474992
*46.* *Lactobacillus.paracasei*	MZ475008
*47.* *Lactobacillus johnsonii*	MZ475009
*48.* *Lactobacillus johnsonii*	MZ474999
*49.* *Enterococcus lactis*	MZ474987
*50.* *Enterococcus lactis*	MZ475006

**Table 5:** Antibiotic pattern profiles of all strains of bacteria (n = 44) isolated from RPL group.

Species * (n)	*E. faecalis* (*20/44*)	*E. faecium* (*6/44*)	*S. haemolyticus* (*8/44*)	*S. aureus* (*2/44*)	*S. caprae* (*1/44*)	*S. hominis* (*1/44*)	*S. epidermidis* (*1/44*)	*Stp. pasteurians* (*1/44*)	*Stp. mitis* (*1/44*)	*Stp.* spp. (*1/44*)	*E. coli* (*1/44*)	*Klebsiella* spp. (*1/44*)	Total Resistance n (%)
OXA (1 µg)	-	-	2 R	2 R	S	R	R	-	-	-	-	-	6 (46.15%)
PG (1 µg)	-	-	-	1 R	-	-	-	S	R	R	-	-	3 (60%)
AMP (2 µg)	7 R	4 R	-	-	-	-	-	-	-	-	R (10 µg)	R (10 µg)	13 (46.42%)
GEN (10 µg)	-	-	5 R	2 R	S	R	R	-	-	-	R	1	11 (73.33%)
GEN HL (30 µg)	9 R	4 R	-	-	-	-	-	R (500 µg)	S (500 µg)	R (500 µg)	-	-	15 (51.72%)
STR HL (300 µg)	12 R	6 R	-	-	-	-	-	R	R	R	-	-	21 (72.41%)
KAN (30 µg)	15 R	3 R	4 R	2 R	S	S	S	R	R	R	S	S	27 (61.36%)
ERY (15 μg)	17 R	5 R	6 R	1 R	R	R	R		R	R	-	-	35 (83.33%)
CLI (2 µg)	-	-	6 R	2 R	S	S	S	R	S	S	-	-	9 (56.25%)
VAN (5 µg)	19 R	3 R	2 R	1 R	S	S	S	S	S	S	-	-	25 (59.52%)
TET (30 µg)	14 R	6 R	4 R	1 R	R	R	R	R	R	R	-	-	31 (73.80%)
CIP (5 µg)	-	-	5 R	2 R	S	R	R	R	S	S	R	R	12 (66.66%)

* E: Enterococcus; S: Staphylococcus; Stp: Streptococcus; S: sensitive; R: resistance; - = not tested; OXA: oxacillin; PG: penicillin G; AMP: ampicillin; GEN: gentamycin; GEN-HL: gentamycin high-level; STR-HL: streptomycin high-level, KAN: kanamycin; ERY: erythromycin; CLI: clindamycin; VAN: vancomycin; TET: tetracycline; CIP: ciprofloxacin.

## Data Availability

The data presented in this study are available on request from the corresponding author. The data are not publicly available due to ownership by the university.

## Supplementary Materials

The following supporting information can be downloaded at: https://www.mdpi.com/article/10.3390/diagnostics12102444/s1, Table S1: Multivariable regression analysis of AV condition with different factors.
