# Supplementary material for: Microbial Diversity and Pathogenic Properties of Microbiota Associated with Aerobic Vaginitis in Women with Recurrent Pregnancy Loss
Source: Diagnostics (Basel). 2022 Oct 9;12(10):2444. doi: 10.3390/diagnostics12102444 (PMC9600244; doi:10.3390/diagnostics12102444)
Supplement: Supplementary file 1 [file diagnostics-12-02444-s001.zip › diagnostics-1920755-supplementary.pdf]

Table S1. Multivariable regression analysis of AV condition with different factors

Variable	odds ratio [OR]	95% [Confidence interval]	Statistical Significance
**Education**	0.73	0.22-2.45	n.s
**Residence**	1.06	0.40-2.82	n.s
**pH**	7.26	2.49-21.19	<0.0001*
**Menstruation**	7.37	2.54-21.38	<0.0001*
